# Brain inflammation is accompanied by peripheral inflammation in *Cstb*^*−/−*^ mice, a model for progressive myoclonus epilepsy

**DOI:** 10.1186/s12974-016-0764-7

**Published:** 2016-11-28

**Authors:** Olesya Okuneva, Zhilin Li, Inken Körber, Saara Tegelberg, Tarja Joensuu, Li Tian, Anna-Elina Lehesjoki

**Affiliations:** 1 Folkhälsan Institute of Genetics, Haartmaninkatu 8, 00014 Helsinki, Finland; 2 Research Program’s Unit, Molecular Neurology, University of Helsinki, Haartmaninkatu 8, 00014 Helsinki, Finland; 3 Neuroscience Center, University of Helsinki, Viikinkaari 4, 00014 Helsinki, Finland; 4 Beijing Huilongguan Hospital, Peking University teaching hospital, Beijing, China

**Keywords:** Cystatin B, Chemokine, CXCL13, Macrophage, M1/M2, Vascularization

## Abstract

Progressive myoclonus epilepsy of Unverricht-Lundborg type (EPM1) is an autosomal recessively inherited childhood-onset neurodegenerative disorder, characterized by myoclonus, seizures, and ataxia. Mutations in the cystatin B gene (*CSTB*) underlie EPM1. The CSTB-deficient (*Cstb* ^*−/−*^) mouse model recapitulates key features of EPM1, including myoclonic seizures. The mice show early microglial activation that precedes seizure onset and neuronal loss and leads to neuroinflammation. We here characterized the inflammatory phenotype of *Cstb* ^*−/−*^ mice in more detail. We found higher concentrations of chemokines and pro-inflammatory cytokines in the serum of *Cstb* ^*−/−*^ mice and higher CXCL13 expression in activated microglia in *Cstb* ^*−/−*^ compared to control mouse brains. The elevated chemokine levels were not accompanied by blood-brain barrier disruption, despite increased brain vascularization. Macrophages in the spleen and brain of *Cstb* ^*−/−*^ mice were predominantly pro-inflammatory. Taken together, these data show that CXCL13 expression is a hallmark of microglial activation in *Cstb* ^*−/−*^ mice and that the brain inflammation is linked to peripheral inflammatory changes, which might contribute to the disease pathology of EPM1.

**Electronic supplementary material:**

The online version of this article (doi:10.1186/s12974-016-0764-7) contains supplementary material, which is available to authorized users.

## Introduction

Progressive myoclonus epilepsy of Unverricht-Lundborg type (EPM1, OMIM 254800) is an autosomal recessively inherited neurodegenerative disorder with onset from 6 to 16 years of age and characterized by action-activated and highly incapacitating myoclonus, tonic-clonic epileptic seizures, and ataxia [1]. EPM1 is caused by loss-of-function mutations in the cystatin B (*CSTB*) gene [2, 3], which encodes an inhibitor of lysosomal cysteine cathepsins [4]. CSTB is highly expressed in immune cells, e.g., in blood leukocytes, hepatic lymphocytes, placental macrophages, and microglia [5–9], and it is upregulated in vitro by pro-inflammatory stimulation [8, 10, 11]. In immune cells, the function of CSTB has been linked to chemotaxis [8], expression and secretion of cytokines, and release of nitric oxide [10, 12, 13], implying a role in the immune response. CSTB function has also been associated with diverse cellular processes, such as regulation of apoptosis [14, 15], bone resorption [16, 17], protection of neurons from oxidative stress [18], and cell cycle progression [19].

A CSTB-deficient mouse model (*Cstb* ^*−/−*^) mimics key features of EPM1, including myoclonic seizures, ataxia [20], and progressive gray and white matter loss [21]. The brain pathology of *Cstb* ^*−/−*^ mice is characterized by microglial activation in asymptomatic mice of 2 weeks of age, followed by widespread activation of astrocytes as well as progressive neuronal death and brain volume loss from 1 month of age onwards [22]. Moreover, activated cultured *Cstb* ^*−/−*^ microglia secrete higher levels of chemokines, such as chemokine (C-C motif) ligand (CCL)2, CCL3, and chemokine (C-X-C motif) ligand (CXCL)1, than control microglia [8]. Gene expression profiling of cultured *Cstb* ^*−/−*^ microglia revealed impaired interferon signaling and also showed altered chemokine expression [23]. Finally, a striking upregulation of *Cxcl13* in gene expression profiling of postnatal day 30 (P30) *Cstb* ^*−/−*^ mouse cerebellum was detected [24].

We here confirm the increased CXCL13 expression also on protein level and show that the inflammatory processes in the *Cstb* ^*−/−*^ brain are linked to peripheral inflammation, which is characterized by increased levels of chemokines and pro-inflammatory cytokines in the serum combined with relatively more pro-inflammatory macrophages, and increased amounts of B lymphocytes in the spleen.

## Materials and methods

### Mice

CSTB-deficient mice (*Cstb* ^*−/−*^) were obtained from The Jackson Laboratory (129-*Cstb* ^tm1Rm^/SvJ; stock no. #003486). Wild-type mice of the same age and background were used as controls. The research protocols were approved by the Animal Ethics Committee of the State Provincial Office of Southern Finland (decision no. ESAVI/7039/04.10.03/2012, ESAVI/5995/04.10.07/2013, and ESAVI/6288/04.10.07/2015).

### Measurement of chemokines and cytokines in mouse serum

Blood samples were obtained by intracardiac puncture of anesthetized P14 and P30 *Cstb* ^*−/−*^ and control mice. The blood was allowed to clot at room temperature (RT) for 15 min and centrifuged at 2000*g* for 13 min. The serum was collected and kept at −80 °C until use. The chemokine and cytokine concentrations were assessed using a combination of mouse CXCL10, interleukin (IL)-1α, CXCL1, IL-6, IL-10, IL-18, IL-1β, IL-12, interferon (IFN)-γ, IFN-α, CCL2, CCL3, CCL4, tumor necrosis factor α (TNFα), colony stimulating factor 2 (GM-CSF), and TGF-β1 FlowCytomix Simplex kits for flow cytometry (eBioscience). The CXCL13 concentration was determined using the Quantikine® mouse CXCL13/BLC/BCA-1 Immunoassay ELISA kit (R&D Systems).

### Tissue processing for histochemical analysis

Anesthetized mice (150 mg/kg pentobarbital) were perfused with phosphate-buffered saline (PBS) (pH 7.4) and 4 % paraformaldehyde (PFA)/PBS for 10 min each. The brains were dissected, immersion fixed in 4 % PFA/PBS for 48 h, and cryoprotected in 30 % sucrose/0.05 % NaN_3_/Tris-buffered saline (TBS) for 3 days. Coronal or sagittal 40-μm sections were cut using a cryostat Leica CM3050 S (Leica Microsystems) and stored in 15 % sucrose/0.05 % NaN_3_/30 % ethylene glycol/TBS.

### Immunohistochemistry

Adjacent 1-in-12 series of coronal free-floating sections (*n* = 5 per genotype and age) were incubated with 50 mM NH_4_Cl for 30 min to reduce non-specific background staining and blocked with 15 % fetal calf serum (FCS) diluted in TBS/0.3 %Triton X-100 (TTX) for 1 h. The sections were incubated with the primary antibodies rabbit anti-ionized calcium-binding adaptor molecule 1 (IBA1; Wako) combined with goat anti-CXCL13, goat anti-CXCL10 (both R&D Systems), or rabbit anti-CXCL1 (Novus Biologicals) in 10 % FCS/TTX for 72 h at 4 °C. The secondary antibodies anti-rabbit Alexa Fluor 488 and anti-goat Alexa Fluor 594 (Invitrogen) were applied for 2 h at RT, and mounted sections were examined using a fluorescence microscope.

### Evaluation of brain vascularity

Histochemical detection of blood vessels was performed as described previously [25]. Adjacent 1-in-12 series of sagittal free-floating sections of non-perfused *Cstb* ^*−/−*^ and control brains (P14 and P30) were incubated in 3,3′-diaminobenzidine (DAB) to detect endogenous peroxidase expression of erythrocytes. From each brain (*n* = 4 per genotype and age), eight sections were analyzed. Per each brain section, the vascularization was quantified from eight black and white bright-field images (×40, five from cortex and three from cerebellum) as relative DAB-positive section area using ImageJ software.

### Measurement of BBB permeability

Blood-brain barrier (BBB) integrity was analyzed based on its permeability for fluorescein [26, 27] and serum albumin [28]. To measure the fluorescein uptake into the brain, *Cstb* ^*−/−*^ and control P30 mice were injected i.p. with 100 μl (5 ml per kg) of 100 mg/ml fluorescein sodium salt (NaF, Sigma-Aldrich) in sterile PBS. After 1 h, the mice were perfused with PBS until the liquid leaving the right atrium was colorless. The excised brains were freed from the meninges and the fourth ventricular choroid plexus and weighed. After homogenization in 500 μl PBS and mixing with a vortex for 2 min, 500 μl of 60 % trichloroacetic acid (Sigma-Aldrich) was added to precipitate protein. Homogenized samples were kept at 4 °C for 30 min and centrifuged at 18,000*g* at 4 °C for 10 min. Fluorescence intensity of the supernatants was measured at excitation 440 nm and emission 525 nm using a microplate reader (WALLAC Victor 2). Fluorescein concentrations were calculated based on a sodium fluorescein standard curve (10 to 200 ng/ml) and expressed as nanogram per milligram brain tissue [29]. For albumin staining, adjacent 1-in-12 series of coronal free-floating sections were incubated with 50 mM NH_4_Cl for 30 min, blocked with 15 % FCS/TTX for 1 h, and incubated for 24 h at 4 °C protected from light with goat anti-mouse FITC-conjugated serum albumin IgG (Alpha Diagnostic International) diluted in 10 % FCS/TTX. Mounted sections were examined using a fluorescence microscope.

### Isolation of brain mononuclear cells and nucleated splenocytes

P14 and P30 mice were euthanized with CO_2_, perfused with ice-cold PBS, and the brain and spleen were dissected. Brain mononuclear cells were isolated as described previously [8]. Splenocytes were collected from spleens by gently grinding through a 40-μm cell strainer, erythrolyzed using VersaLyse lysing solution (Beckman Coulter), and washed with ice-cold PBS.

### Flow cytometry

The above isolated cells were blocked with 10 % normal rat serum/PBS on ice for 30 min. The brain mononuclear cells were stained with a combination of anti-mouse antibodies CD206-FITC + MHCII-PE + F4/80-PE/Cy7 + CD45-APC and the splenocytes with a combination of CD11b-FITC + CD45-PE + F4/80-PE/Cy7 + Gr-1-APC or CD206-FITC + MHCII-PE + F4/80-PE/Cy7 + CD45-APC (all from BioLegend) on ice, protected from light, for 30 min. Cells were washed and resuspended in 500 μl PBS/1 % FCS/0.02 % NaN_3_. The flow cytometric data were acquired with a two-laser, six-color Gallios flow cytometer and analyzed by Kaluza analysis 1.3 software (Beckman Coulter). Brain mononuclear cells were defined as follows: microglia CD45^+^F4/80^+^, macrophages CD45^hi^F4/80^+^, M1-type macrophages CD45^hi^F4/80^+^MHCII^+^CD206^−^, and M2-type macrophages CD45^hi^F4/80^+^MHCII^−/+^CD206^+^. Splenocytes were defined as follows: granulocytes CD45^+^F4/80^−/+^Gr-1^++^, monocytes CD45^+^F4/80^−^Gr-1^+^, monocyte-derived macrophages CD45^+^CD11b^+^F4/80^+^Gr-1^−^, tissue-resident macrophages CD45^+^F4/80^++^Gr-1^−/+^, M1-type macrophages CD45^+^F4/80^+^MHCII^+^CD206^−^, and M2-type macrophages CD45^+^F4/80^+^MHCII^−/+^CD206^+^. Cell populations were calculated as percentages among total leukocytes or macrophages.

### Statistical analyses

Statistical analyses were performed using unpaired, two-sided *t* test or two-way analysis of variance (ANOVA) test with Sidak’s multiple comparison test for comparison between genotypes. All data are presented as mean ± SEM and a value of *p* < 0.05 is considered statistically significant.

Further methods are available in the Supporting Information (Additional file 1).

## Results

### Pro-inflammatory cytokine levels are high in the serum of young *Cstb*^*−/−*^ mice

**Fig. 1:**
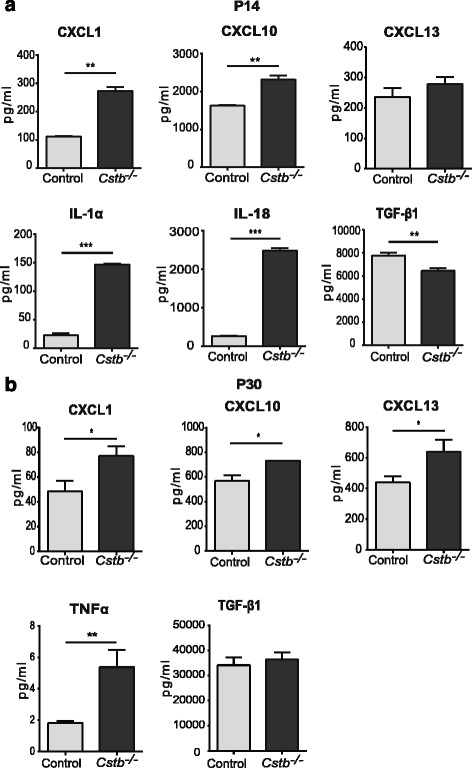
Cytokine levels in the serum of control and *Cstb* ^*−/−*^ mice. **a** Concentrations of CXCL1, CXCL10, CXCL13, IL-1α, IL-18, and TGF-β1 at P14 and **b** CXCL1, CXCL10, CXCL13, TNF-α, and TGF-β1 at P30. Data are presented as mean ± SEM (*n* = 3–6 per genotype; *p < 0.05, **p < 0.01, ***p < 0.001)

To characterize peripheral inflammatory changes in pre-symptomatic and early symptomatic *Cstb* ^*−/−*^ mice, we determined the concentrations of 17 cytokines and chemokines in the serum of *Cstb* ^*−/−*^ and control mice at P14 and P30. At P14, the concentrations of pro-inflammatory chemokines CXCL1 and CXCL10, as well as pro-inflammatory cytokines IL-1α and IL-18, were significantly higher in *Cstb* ^*−/−*^ than in control mice (Fig. 1a). In contrast, the concentration of anti-inflammatory cytokine TGF-β1 was reduced. The levels of CXCL1, CXCL10, and TNF-α were higher in the serum of P30 *Cstb* ^*−/−*^ than in control mice, whereas the level of TGF-β1 did not differ between genotypes (Fig. 1b). The level of CXCL13 did not differ at P14, but was increased at P30. In conclusion, these data imply the presence of systemic inflammation, characterized by increased level of chemokines and pro-inflammatory cytokines already in pre-symptomatic *Cstb* ^*−/−*^ mice at P14.

### Expression of the pro-inflammatory chemokine CXCL13 is highly increased in *Cstb*^*−/−*^ microglia

**Fig. 2:**
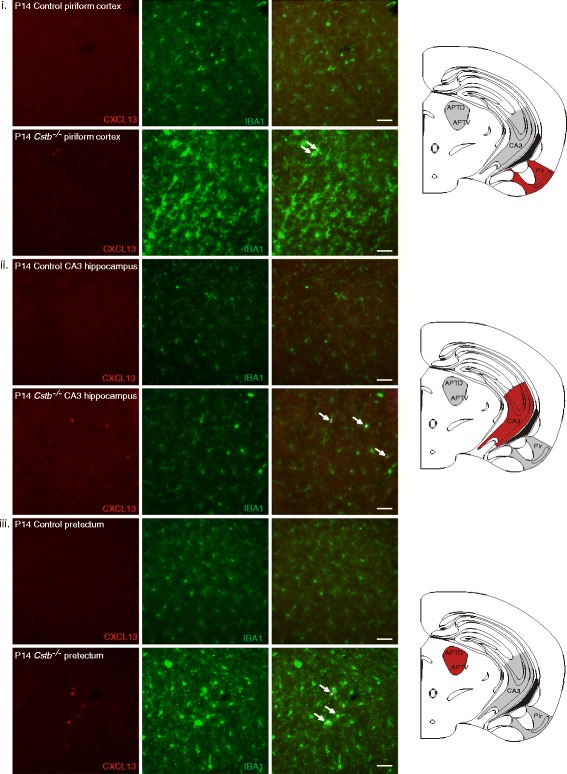
Immunohistochemical detection of CXCL13 in control and *Cstb* ^*−/−*^ mouse brain at P14. CXCL13-positive microglia are shown by double immunofluorescence staining of CXCL13 (*red*) with the microglial marker IBA1 (*green*) in the following brain areas: **i** piriform cortex, **ii** CA3 area of the hippocampus, and **iii** pretectum of control and *Cstb* ^*−/−*^ mice. Representative CXCL13- and IBA1-double-positive cells in the merged image are marked with arrows. *Scale bar* = 50 μM

**Fig. 3:**
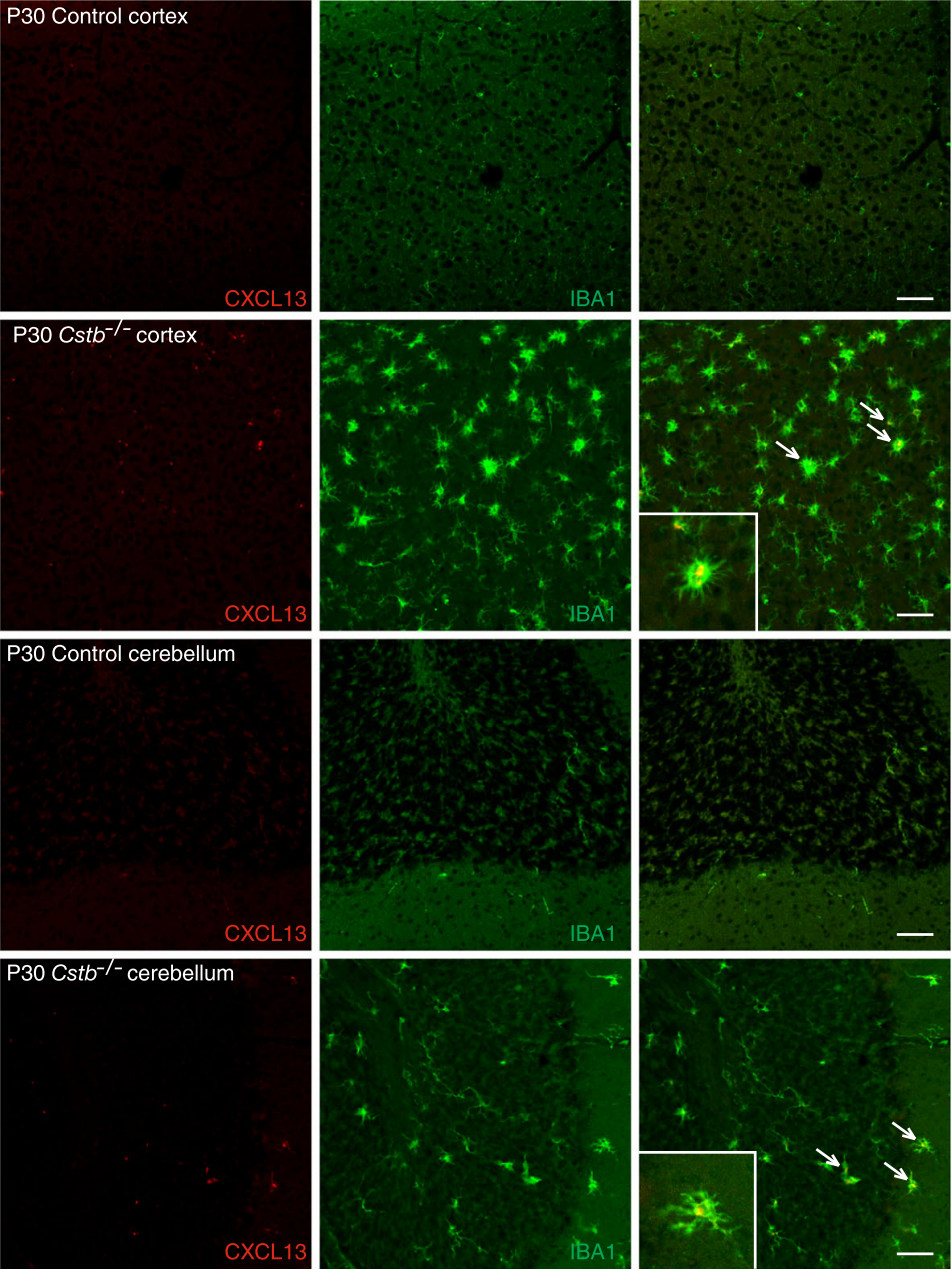
Immunohistochemical detection of CXCL13 in control and *Cstb* ^*−/−*^ mouse brain at P30. CXCL13-positive microglia are shown by double immunofluorescence staining of CXCL13 (*red*) with the microglial marker IBA1 (*green*) in the cortex and cerebellum of control and *Cstb* ^*−/−*^ mice. Representative CXCL13- and IBA1-double-positive cells in the merged image are marked with *arrows*. The *inserts* show enlargements of one double immuno-positive cell from both brain regions. *Scale bar* = 50 μM

As the expression and secretion of chemokines have previously been shown to be altered in cerebellar tissue and primary microglia of *Cstb* ^*−/−*^ mice [8, 23, 24], we focused our further analyses on brain expression of chemokines CXCL1, CXCL10, and CXCL13, which were increased in the sera of mice at P30. Using immunohistochemistry in *Cstb* ^*−/−*^ and control mice, we did not detect expression of CXCL1 and only low level of CXCL10 at P14 and P30 (data not shown). Expression of CXCL13 was higher in *Cstb* ^*−/−*^ than control brain at both time points (Figs. 2 and 3). In P14 *Cstb* ^*−/−*^ brain tissue, CXCL13 immunopositivity was restricted to the piriform cortex, the CA3 area of the hippocampus, and the dorsal and ventral part of the anterior pretectal nucleus (Fig. 2), whereas the other cortical areas or the cerebellum did not express CXCL13 (data not shown). At P30, CXCL13 was highly expressed also in other regions of the cortex and in the cerebellum (Fig. 3). CXCL13 immunopositivity co-localized with IBA1 immunopositivity, marking *Cstb* ^*−/−*^ microglia that have an activated morphology.

### Brain vascularization is enhanced and the BBB is intact in young *Cstb*^*−/−*^ mice

**Fig. 4:**
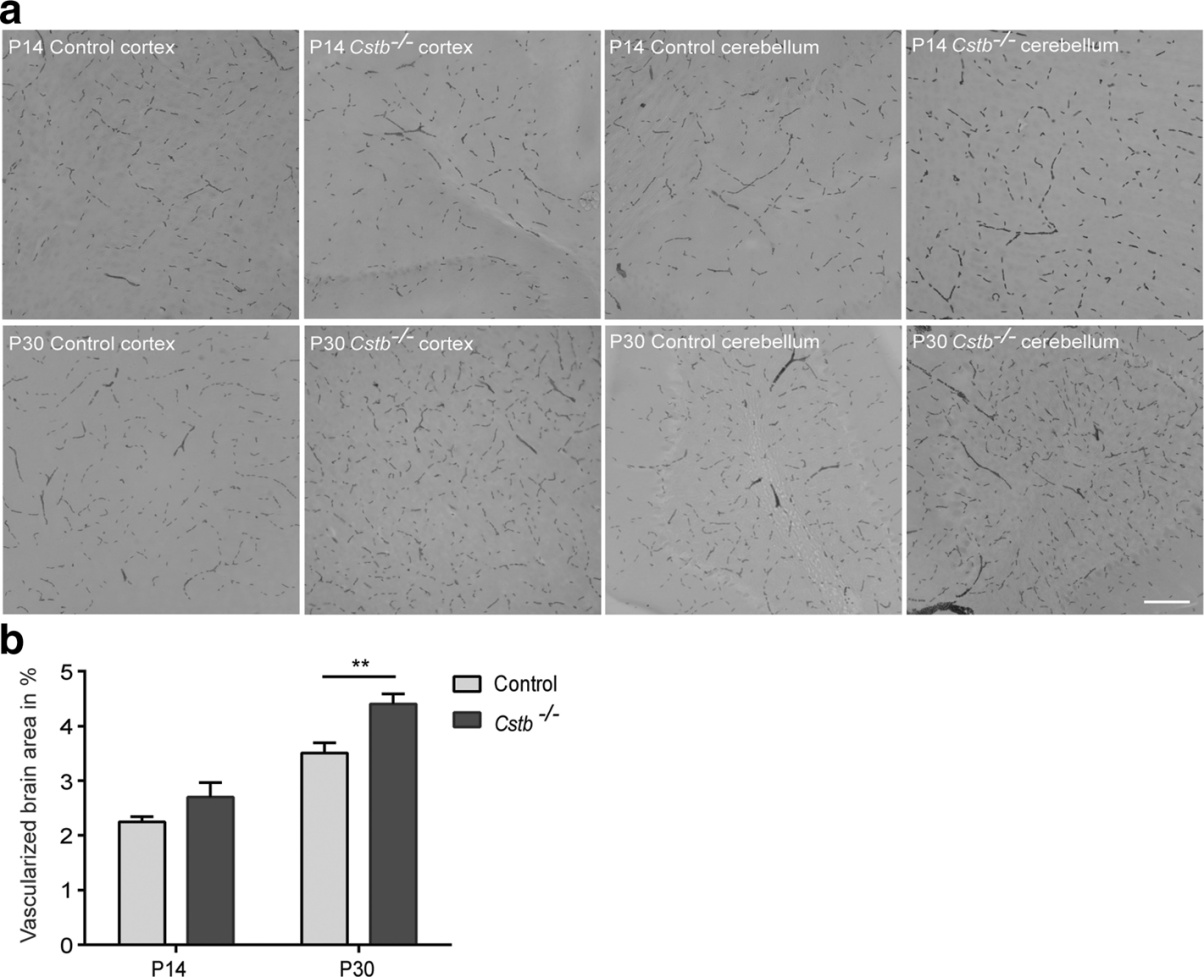
Brain vascularization of control and *Cstb* ^*−/−*^ mice. **a** Histochemical detection of brain vessels in the cortex of control and *Cstb* ^*−/−*^ mice at P14 and P30 was performed using DAB, which detects erythrocytes based on their endogenous peroxidase expression. **b** Vascularization is quantified at P14 and at P30 as relative DAB-positive area in 64 images from each of four control and four *Cstb* ^*−/−*^ brains. Data are presented as mean ± SEM (**p < 0.01, *scale bar* = 50 μM)

Chemokines are involved in the regulation of angiogenesis [30]. Therefore, we analyzed the vascularization in *Cstb* ^*−/−*^ and control mice at P14 and P30 in non-perfused brains by determining the relative area positive for histochemical DAB staining, which detects endogenous erythrocyte peroxidase (Fig. 4a). At P14, the extent of brain vascularization did not differ significantly between genotypes, but it was more intense in *Cstb* ^*−/−*^ than in control mice at P30 (Fig. 4b). To determine whether this increased vascularization is associated with higher BBB permeability, we measured the BBB integrity based on the presence of peripherally injected sodium fluorescein or endogenous serum albumin in the brain tissue at P30. Neither method revealed differences in BBB permeability between *Cstb* ^*−/−*^ and control mice (Additional file 2: Figure S1).

### Macrophages are pro-inflammatory in *Cstb*^*−/−*^ mice

**Fig. 5:**
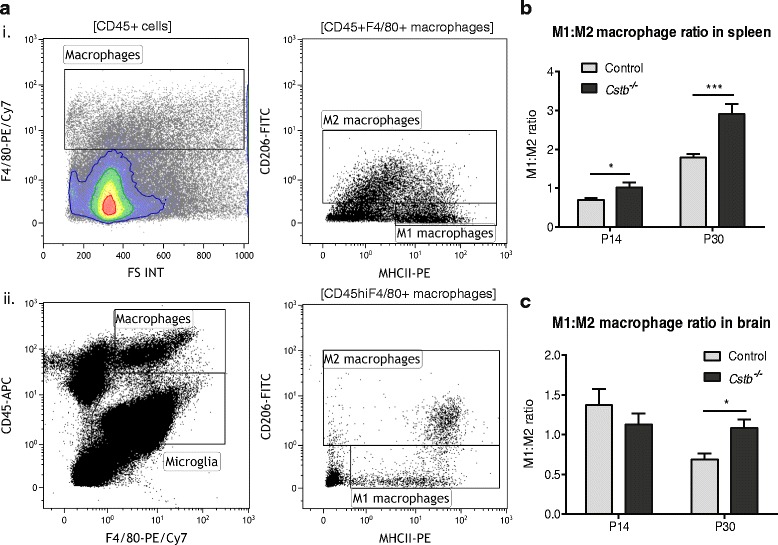
Flow cytometric analysis of M1 and M2 macrophages in control and *Cstb* ^*−/−*^ spleen and brain. **a** Illustrative plots show the flow cytometric gating strategy of nucleated spleen cells and enriched brain mononuclear cells. (*i*) In the spleen, CD45^+^F4/80^+^ macrophages were divided into CD45^+^F4/80^+^MHCII^+^CD206^−^ M1 and CD45^+^F4/80^+^MHCII^−/+^CD206^+^ M2 cells. (*ii*) In the brain, the CD45^hi^F4/80^+^ macrophage population was divided into CD45^hi^F4/80^+^MHCII^+^CD206^−^ M1 and CD45^hi^F4/80^+^MHCII^−/+^CD206^+^ M2 macrophages. Ratio between M1 and M2 macrophages at P14 and P30 (M1:M2 ratio) in the **b** spleen (*n* = 6 samples) and **c** brain (*n* = 15 samples per genotype at P14 and *n* = 11 per genotype at P30). Data are presented as mean ± SEM (*p < 0.05, ***p < 0.001)

To determine whether the high levels of pro-inflammatory cytokines in the serum are associated with changes in immune cell populations, we performed flow cytometric analyses to characterize the composition and activation of different immune cell types in *Cstb* ^*−/−*^ mouse bone marrow, spleen, and brain at P14 and P30. First, we determined the myeloid cell composition in the spleen and bone marrow (Additional file 3: Figure S2), as well as granulocyte-macrophage and macrophage-dendritic cell progenitors in the bone marrow (Additional file 4: Figure S3). We did not detect any differences between genotypes at either time point. In addition, because CXCL13 is a chemoattractant for B lymphocytes [31, 32], and it is highly expressed at P30, we determined the relative amount of B lymphocytes among brain, spleen, and bone marrow leukocytes at P30 (Additional file 5: Figure S4A). It was significantly higher in the spleen (Additional file 5: Figure S4B), but did not differ in the brain and bone marrow between genotypes (Additional file 5: Figure S4C, D). Finally, we characterized the immune phenotype of spleen and brain macrophages by specifying the relative amount of pro-inflammatory M1 and anti-inflammatory M2 macrophages from the total amount of macrophages in each tissue (Fig. 5a and Additional file 6: Figure S5) and determined the ratio (M1:M2) between both types. The ratio was higher in *Cstb* ^*−/−*^ mice than in controls at P14 and P30 in the spleen (Fig. 5b). In the brain of P30 *Cstb* ^*−/−*^ mice, the macrophages were also more polarized towards the pro-inflammatory M1 type than control macrophages (Fig. 5d).

## Discussion

In this study, we show that altered levels of chemokines in the serum and brain of young *Cstb* ^*−/−*^ mice, which indicate systemic inflammation already in pre-symptomatic mice, is linked to increased brain vascularization in the presence of a seemingly intact BBB. Moreover, we show that high CXCL13 expression is a hallmark of activated *Cstb* ^*−/−*^ microglia and that macrophages in the *Cstb* ^*−/−*^ spleen and brain are pro-inflammatory.

Traumatic brain injury, epileptic seizures, ischemia, multiple sclerosis, and neurodegenerative diseases, which are characterized by a higher prevalence or a reduced threshold for seizures, are all associated with the expression and secretion of cytokines [33, 34]. Cytokines and chemokines are released primarily by cells of the immune system and vascular endothelial cells, and they can actively cross the BBB or stimulate endothelial cells to express mediators that activate brain cells [35, 36]. Previously, it had been shown that the levels of pro-inflammatory cytokines IL-18, IL-1β, and TNFα are increased in the serum of adult *Cstb* ^*−/−*^ mice after peripheral LPS injection [12]. Interestingly, we identified elevated levels of IL-18 and TNFα already in young *Cstb* ^*−/−*^ mice without activation of inflammation with LPS, whereas no alterations in the level of IL-1β were seen.

We also identified increased serum levels of chemokine CXCL1, CXCL10, and CXCL13 in *Cstb* ^*−/−*^ mice. Whether these chemokines are secreted from immune cells or endothelial cells requires further studies. Expression of CXCL13, which binds CXCR4 receptor and regulates B cell migration [32], has been reported to be enhanced in inflammatory CNS diseases, such as multiple sclerosis and encephalitis [37–40]. Our previous gene expression analysis of P30 *Cstb* ^*−/−*^ cerebellar tissue revealed a striking (29-fold) upregulation of *Cxcl13* [24]. On the contrary, in transcriptomics profiling of in vitro-cultured *Cstb* ^*−/−*^ microglia, a slight downregulation of *Cxcl13* was observed [23], suggesting that the CXCL13 upregulation in *Cstb* ^*−/−*^ microglia might be specific to the brain in vivo. In line with other studies, which have shown CXCL13 expression in activated mouse microglia and in blood-derived human monocytes and macrophages [41–44], we detected increased expression of CXCL13 in IBA1-positive microglia. Therefore, CXCL13 serves as a marker for activated microglia in *Cstb* ^*−/−*^ mice. Interestingly, the expression of CXCL13 at P14 in *Cstb* ^*−/−*^ microglia was restricted to the piriform cortex, CA3 area of the hippocampus, and pretectum, but was more widespread at P30.

Chemokines can regulate the integrity of the BBB [45, 46]. In particular, they affect angiogenesis and BBB permeability [30, 45, 46]. The chemokines CXCL10 and CXCL13 have been reported to be angiostatic, i.e., inhibiting the generation of vessels, whereas CXCL1 is angiogenic inducing vessel formation [30, 47]. Our results imply more intense brain vascularization in P30 *Cstb* ^*−/−*^ mice, which might be mediated by the higher CXCL1 concentration in serum. In addition, CXCL10 and CXCL13 could be upregulated in the serum and in the brain, respectively, to counteract the angiogenic effect of CXCL1. Despite the elevated levels of cytokines in the serum of *Cstb* ^*−/−*^ mice and the previously shown higher presence of macrophages, T cells, and granulocytes in the brain [8], we did not detect a compromised BBB yet at P30. However, it is likely that the BBB integrity will be impaired in older *Cstb* ^*−/−*^ mice as a consequence of a prolonged inflammation in the brain.

Although CXCL13 has been reported to function as a B cell chemoattractant [31, 32], we did not detect a greater proportion of B cells in the *Cstb* ^*−/−*^ brain. In line with our finding, the B cell infiltration after experimental autoimmune encephalomyelitis has been shown to be normal in the brain of CXCL13-deficient mice [48]. We did find an increased B cell population in the spleen of *Cstb* ^*−/−*^ mice, but the mechanism and significance of this finding warrant further studies.

In response to inflammatory stimuli or pathogens, microglia and macrophages can be broadly classified into pro- (M1) or anti-inflammatory (M2) activated [49–51]. Pro-inflammatory activation is linked to the release of pro-inflammatory cytokines and mediators, whereas anti-inflammatory cells promote tissue repair and survival. However, microglia and macrophages adopt various intermediate phenotypes in vivo depending on the nature of the activating stimuli. Therefore, the M1-M2 classification does not reflect the full spectrum of the intermediate and mutually non-exclusive “activation” states in vivo. A recent report by Murray et al. [52] revised the nomenclature for macrophages in vitro based on the activating stimuli. In relation to this framework, the M1 population in our study, which we identified based on their low mannose receptor (CD206) and high MHCII expression level, can be related to the M(IFN-γ) population because MHCII expression is induced by IFN-γ [53, 54]. Moreover, the M2 population, which we defined based on their high CD206 expression level, can be related to M(IL-4) cells because IL-4 stimulation induces CD206 upregulation [52, 55]. Using flow cytometric analysis, we previously showed that microglia directly extracted from the brain are skewed towards the anti-inflammatory phenotype in P14 and towards the pro-inflammatory phenotype in P30 *Cstb* ^*−/−*^ mice [8]. In line with these findings, also, splenic and brain macrophages show a prevailing pro-inflammatory, M1-type polarization at P30. These data imply that not only microglia but also macrophage cell populations contribute to the emergence of brain and peripheral inflammation.

In conclusion, our results support the previously described association of CSTB deficiency with early inflammatory processes in the brain of *Cstb* ^*−/−*^ mice. Here, we report altered chemokine and cytokine level in the serum of *Cstb* ^*−/−*^ mice. Future studies will show whether these findings recapitulate in EPM1 patients and whether altered expression of chemokines and/or cytokines could be useful biomarkers for diagnosis, prognosis, and treatment efficacy. Increased understanding of the inflammatory mechanisms in EPM1 is a prerequisite for the development of novel therapeutic strategies to treat this devastating disease.

## Additional files

Additional file 1: Flow cytometric analysis of lymphocytes, bone marrow cells, and bone marrow progenitors. (PDF 86 kb)
Additional file 2: Figure S1. BBB permeability of control and *Cstb* ^*−/−*^ mice. (A) Levels of sodium fluorescein (NaF) in the brain of control and *Cstb* ^*−/−*^ mice at P30 (*n* = 5 per genotype). (B) Immunohistochemical detection of albumin-FITC (green) in the brain of control and *Cstb* ^*−/−*^ mice at P30 (red: microglial marker IBA1, *n* = 4 per genotype). Data are presented as mean ± SEM (n.s.—statistically not different, scale bar = 50 μM). (PDF 5545 kb)
Additional file 3: Figure S2. Flow cytometric analysis of myeloid cells from control and *Cstb* ^*−/−*^ mouse spleen and bone marrow. (A) Illustrative plots show the flow cytometric gating strategy of enriched nucleated cells from spleen and bone marrow. In spleen, the CD45^+^ leukocytes were divided into (i) CD45^+^F4/80^−/+^Gr-1^++^ granulocytes, CD45^+^F4/80^−^Gr-1^+^ monocytes, CD45^+^F4/80^++^Gr-1^−/+^ tissue-resident macrophages and (ii) CD45^+^CD11b^+^F4/80^+^Gr-1^−^ monocyte-derived macrophages. In the bone marrow, the CD45^+^ leukocytes were divided into (iii) CD45^+^F4/80^−/+^Gr-1^++^ granulocytes, CD45^+^F4/80^−^Gr-1^+^ monocytes, and CD45^+^F4/80^+^Gr-1^−/+^ macrophages. (B) Percentages of granulocytes, monocytes, and tissue-resident and monocyte-derived macrophages in the total CD45^+^ leukocyte population in spleen of control and *Cstb* ^*−/−*^ mice at P14 and P30. (C) Percentages of granulocytes, monocytes, and macrophages in the total CD45^+^ leukocyte population in the bone marrow of control and *Cstb* ^*−/−*^ mice at P14 and P30. Data are presented as mean ± SEM (*n* = 15 samples per genotype at P14, and *n* = 11 samples per genotype at P30). (PDF 1769 kb)
Additional file 4: Figure S3. Analysis of granulocyte-macrophage progenitors (GMP) and macrophage-dendritic cell progenitors (MDP) in control and *Cstb* ^*−/−*^ mice. (A) Illustrative plots show the flow cytometric gating strategy of progenitor cells from bone marrow. GMP cells are represented as Lin^−^c-Kit^+^Sca-1^−^CD16/32^+^CD115^−^ and MDP cells as Lin^−^c-Kit^+^Sca-1^−^CD16/32^+^CD115^+^. Percentages of (B) GMP and (C) MDP cells in the total bone marrow leucocytes of control and *Cstb* ^*−/−*^ mice at P14 and P30. Data are presented as mean ± SEM (*n* = 6 samples per genotype; each sample containing cells from one mouse). (PDF 1712 kb)
Additional file 5: Figure S4. Analysis of B lymphocytes from the spleen, brain, and bone marrow of control and *Cstb* ^*−/−*^ mice. (A) Illustrative plots show the flow cytometric gating strategy of lymphocytes from the (i) spleen, (ii) brain, and (iii) bone marrow. Percentage of B lymphocytes in the total CD45^+^ cell population in the (B) spleen, (C) brain, and (D) bone marrow of control and *Cstb* ^*−/−*^ mice at P30. Data are presented as mean ± SEM (*n* = 5 samples per genotype; each sample containing cells from one mouse; ****p* < 0.001). (PDF 3206 kb)
Additional file 6: Figure S5. Flow cytometric analysis of M1 and M2 macrophages in control and *Cstb* ^*−/−*^ mouse spleen and brain. Percentages of M1 and M2 macrophages in the total macrophage population in the (A and B) spleen and (C and D) brain of control and *Cstb* ^*−/−*^ mice at P14 and P30. (**p* < 0.05, ***p* < 0.01, ****p* < 0.001). (PDF 309 kb)

## Abbreviations

BBB: Blood-brain barrier
CSTB: Cystatin B
EPM1: Progressive myoclonus epilepsy of Unverricht-Lundborg type
