# Supplementary material for: Brain inflammation is accompanied by peripheral inflammation in Cstb−/− mice, a model for progressive myoclonus epilepsy
Source: J Neuroinflammation. 2016 Nov 28;13:298. doi: 10.1186/s12974-016-0764-7 (PMC5127053; doi:10.1186/s12974-016-0764-7)
Supplement: Additional file 1: — Flow cytometric analysis of lymphocytes, bone marrow cells, and bone marrow progenitors. (PDF 86 kb) [file 12974_2016_764_MOESM1_ESM.pdf]

Flow cytometric analysis of lymphocytes, bone marrow cells, and bone marrow progenitors

P14 and P30 mice were euthanized with CO_2 and perfused with ice-cold PBS. Tissues including brain, bone marrow, and spleen were then dissected. The brains were gently homogenized through 70 µm cell strainers (Fisher Scientific) with 7 ml of RPMI 1640 medium (Lonza) supplemented with 2 mM EDTA. 3 ml of 100% isotonic Percoll (GE Healthcare) was added into the cell suspension to make a final 30% isotonic Percoll, which was then carefully layered on top of 70% isotonic Percoll. The gradient was then centrifuged at 500×g for 30 min without brake. Mononuclear cells were collected from the interphase of Percoll layers and washed with PBS. Bone marrow cells from both femurs were flushed out with PBS using a 22-gauge needle and centrifuged at 2000 rpm for 5 min. Spleens were gently grinded through 40 µm cell strainers. The femoral bone marrow cells and splenocytes were then erythrolysed using Versalyse solution (Beckman Coulter) and washed with ice-cold PBS. The isolated cells were blocked with 10% normal rat serum in ice-cold PBS for 30 min.

The bone marrow cells were stained with CD11b-FITC + CD45-PE + F4/80-PE/Cy7 + Gr-1-APC (all from BioLegend) on ice, protected from light, for 30 min. Cells were washed and resuspended into 500 µl PBS/1% FCS/0.02% NaN_3. The flow cytometric data were acquired with a 2-laser, 6-color Gallios flow cytometer and analyzed by Kaluza analysis 1.3 software (Beckman Coulter). Bone marrow nucleated cells were defined as follows: granulocytes $CD45^{+}F4/80^{-/+}Gr\text{-}1^{++}$, monocytes $CD45^{+}F4/80^{-}Gr\text{-}1^{+}$, and macrophages $CD45^{+}F4/80^{+}Gr\text{-}1^{-/+}$. Cell populations were calculated as percentages of total leukocytes or macrophages.

The isolated cells were blocked with 10% normal rat serum in ice-cold PBS for 30 min. The brain mononuclear cells were stained with CD3-FITC + CD45-PE + CD8-PE/Cy5.5 + CD4-PE/Cy7 +

CD19-APC, the bone marrow cells with CD3-FITC + CD45-PE + CD8-PE/Cy5.5 + CD4-PE/Cy7 + CD19-APC or LIN-FITC + Sca1-PE + CD16/32-PE/Cy5.5 + cKit-PE/Cy7 + CD115-APC, and the splenocytes with CD3-FITC + CD45-PE + CD8-PE/Cy5.5 + CD4-PE/Cy7 + CD19-APC (all from BioLegend) on ice protected from light for 30 min. Cells were washed and resuspended into 500 µl PBS/1% FCS/0.02% NaN_3 for flow cytometric analysis. Samples were acquired with a 2-laser, 6-col-or Gallios flow cytometer and analyzed by Kaluza analysis 1.3 software (Beckman Coulter). In the bone marrow granulocyte-macrophage progenitor (GMP) and common macrophage and dendritic cell precursor (MDP) cells were defined as $Lin^{-}c\text{-}Kit^{+}Sca\text{-}1^{-}CD16/32^{+}CD115^{-}$ and $Lin^{-}c\text{-}Kit^{+}Sca\text{-}1^{-}CD16/32^{+}CD115^{+}$ cells, respectively. B lymphocytes in the brain, bone marrow and spleen were defined as $CD45^{+}CD19^{+}$ cells. Cell populations were calculated as the percentages among total leukocytes.
