# Supplementary figures and images for: Brain inflammation is accompanied by peripheral inflammation in Cstb−/− mice, a model for progressive myoclonus epilepsy
Source: J Neuroinflammation. 2016 Nov 28;13:298. doi: 10.1186/s12974-016-0764-7 (PMC5127053; doi:10.1186/s12974-016-0764-7)

**A**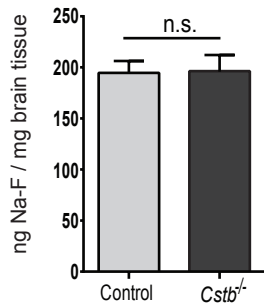**B**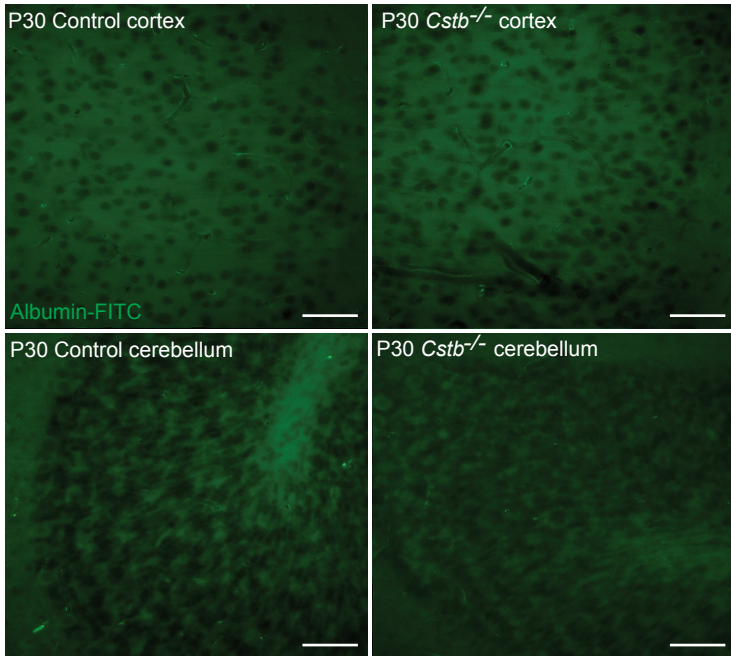

Supplement: Additional file 2: Figure S1. — BBB permeability of control and Cstb −/− mice. (A) Levels of sodium fluorescein (NaF) in the brain of control and Cstb −/− mice at P30 (n = 5 per genotype). (B) Immunohistochemical detection of albumin-FITC (green) in the brain of control and Cstb −/− mice at P30 (red: microglial marker IBA1, n = 4 per genotype). Data are presented as mean ± SEM (n.s.—statistically not different, scale bar = 50 μM). (PDF 5545 kb) [file 12974_2016_764_MOESM2_ESM.pdf]

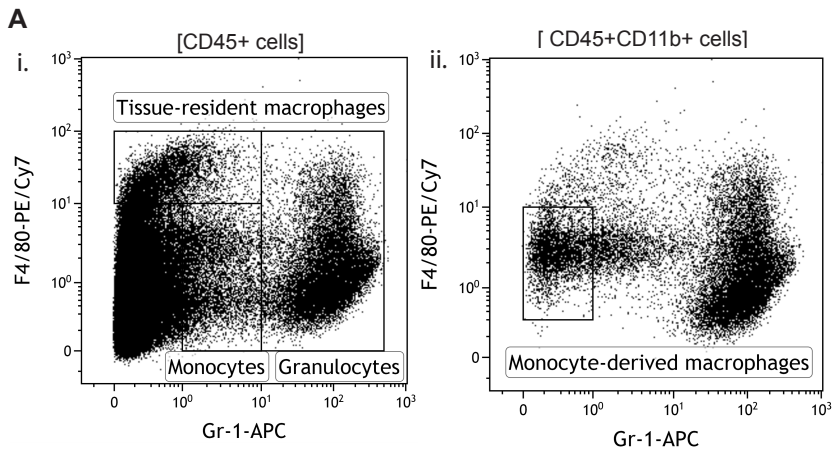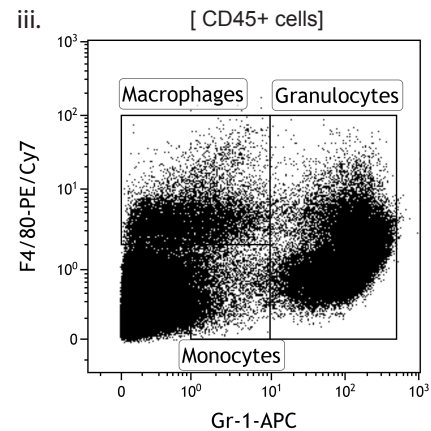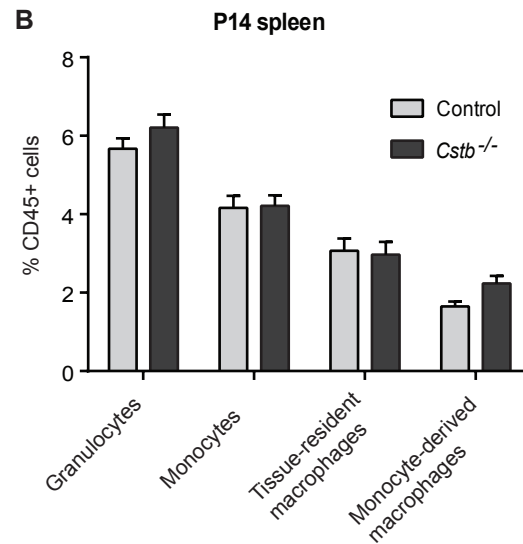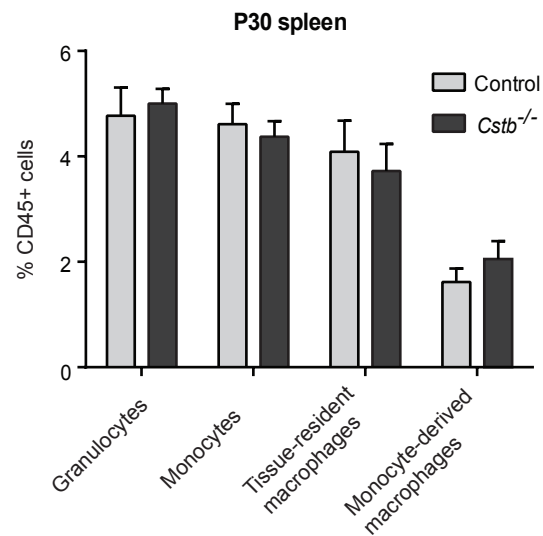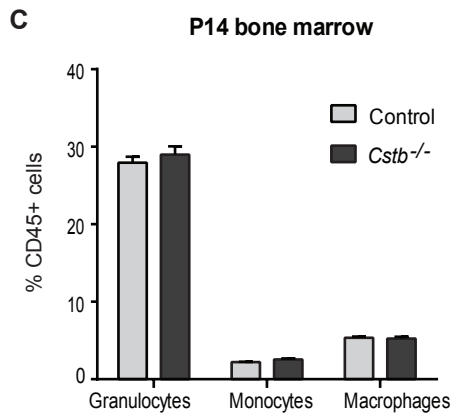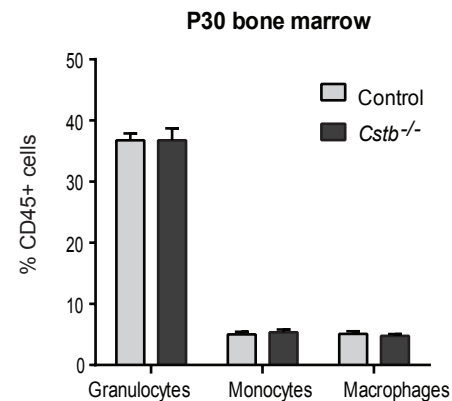

Supplement: Additional file 3: Figure S2. — Flow cytometric analysis of myeloid cells from control and Cstb −/− mouse spleen and bone marrow. (A) Illustrative plots show the flow cytometric gating strategy of enriched nucleated cells from spleen and bone marrow. In spleen, the CD45+ leukocytes were divided into (i) CD45+F4/80−/+Gr-1++ granulocytes, CD45+F4/80−Gr-1+ monocytes, CD45+F4/80++Gr-1−/+ tissue-resident macrophages and (ii) CD45+CD11b+F4/80+Gr-1− monocyte-derived macrophages. In the bone marrow, the CD45+ leukocytes were divided into (iii) CD45+F4/80−/+Gr-1++ granulocytes, CD45+F4/80−Gr-1+ monocytes, and CD45+F4/80+Gr-1−/+ macrophages. (B) Percentages of granulocytes, monocytes, and tissue-resident and monocyte-derived macrophages in the total CD45+ leukocyte population in spleen of control and Cstb −/− mice at P14 and P30. (C) Percentages of granulocytes, monocytes, and macrophages in the total CD45+ leukocyte population in the bone marrow of control and Cstb −/− mice at P14 and P30. Data are presented as mean ± SEM (n = 15 samples per genotype at P14, and n = 11 samples per genotype at P30). (PDF 1769 kb) [file 12974_2016_764_MOESM3_ESM.pdf]

**A**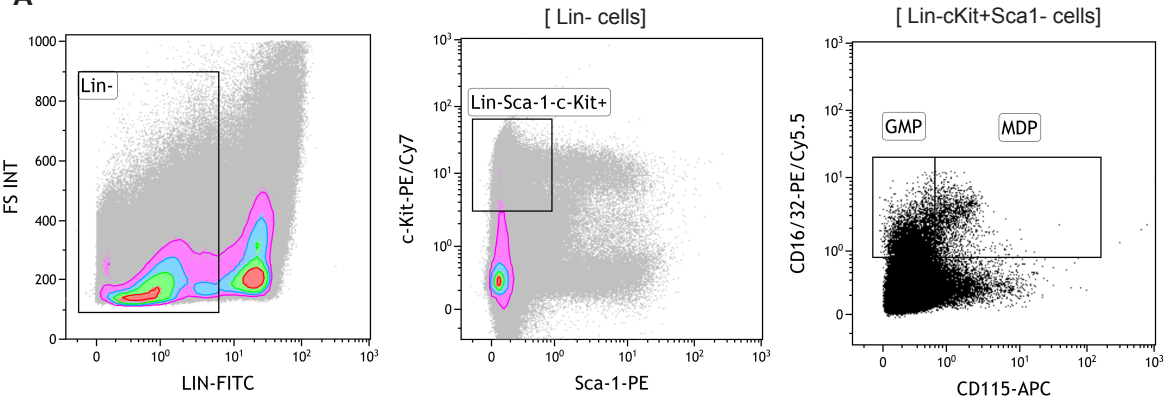**B**

granulocyte-macrophage  
progenitors (GMP)

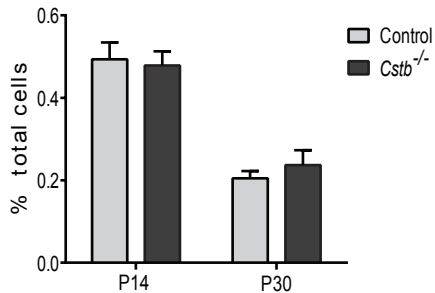**C**

macrophage-dendritic cell  
progenitors (MDP)

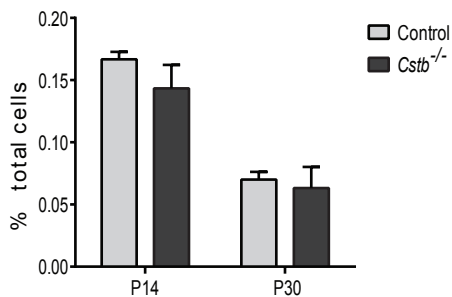

Supplement: Additional file 4: Figure S3. — Analysis of granulocyte-macrophage progenitors (GMP) and macrophage-dendritic cell progenitors (MDP) in control and Cstb −/− mice. (A) Illustrative plots show the flow cytometric gating strategy of progenitor cells from bone marrow. GMP cells are represented as Lin−c-Kit+Sca-1−CD16/32+CD115− and MDP cells as Lin−c-Kit+Sca-1−CD16/32+CD115+. Percentages of (B) GMP and (C) MDP cells in the total bone marrow leucocytes of control and Cstb −/− mice at P14 and P30. Data are presented as mean ± SEM (n = 6 samples per genotype; each sample containing cells from one mouse). (PDF 1712 kb) [file 12974_2016_764_MOESM4_ESM.pdf]

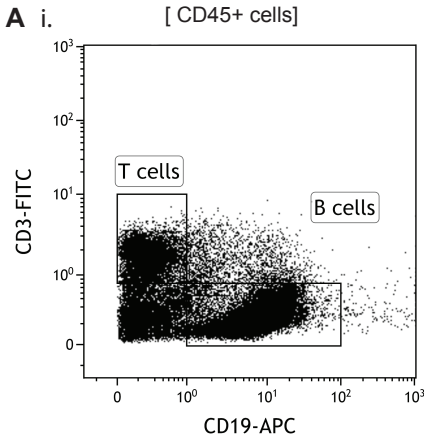

**B**

### B lymphocytes in P30 spleen

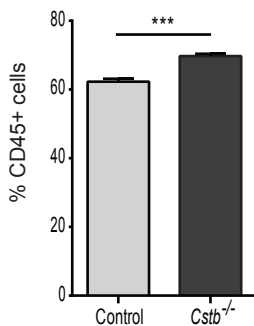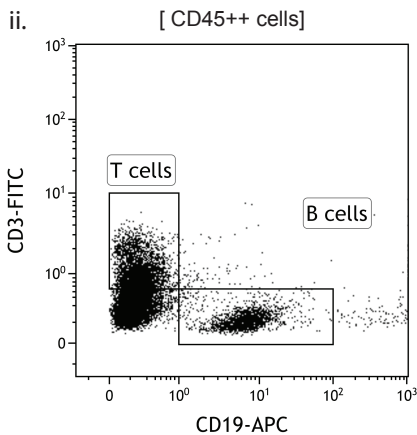

**C**

### B lymphocytes in P30 brain

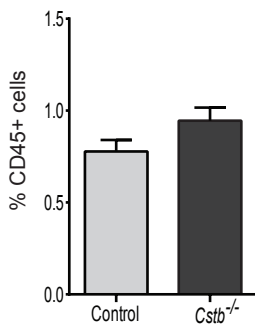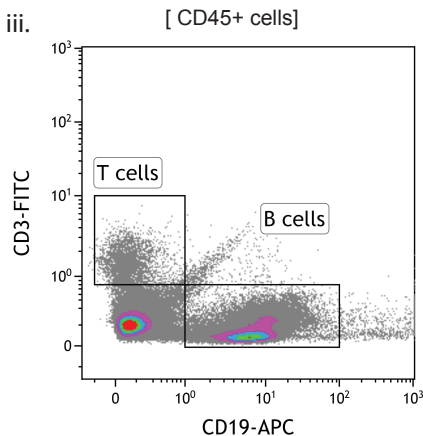

**D**

### B lymphocytes in P30 bone marrow

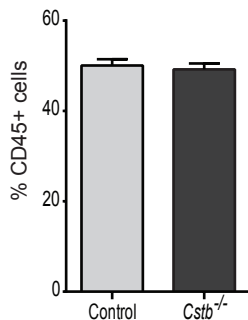

Supplement: Additional file 5: Figure S4. — Analysis of B lymphocytes from the spleen, brain, and bone marrow of control and Cstb −/− mice. (A) Illustrative plots show the flow cytometric gating strategy of lymphocytes from the (i) spleen, (ii) brain, and (iii) bone marrow. Percentage of B lymphocytes in the total CD45+ cell population in the (B) spleen, (C) brain, and (D) bone marrow of control and Cstb −/− mice at P30. Data are presented as mean ± SEM (n = 5 samples per genotype; each sample containing cells from one mouse; ***p < 0.001). (PDF 3206 kb) [file 12974_2016_764_MOESM5_ESM.pdf]

**A****P14 spleen**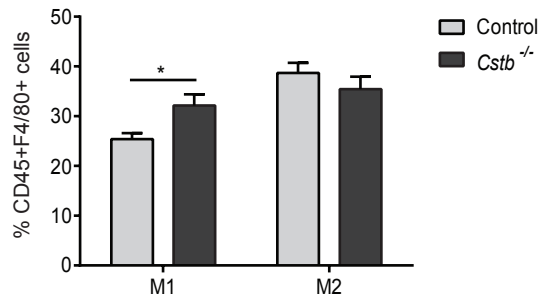**B****P30 spleen**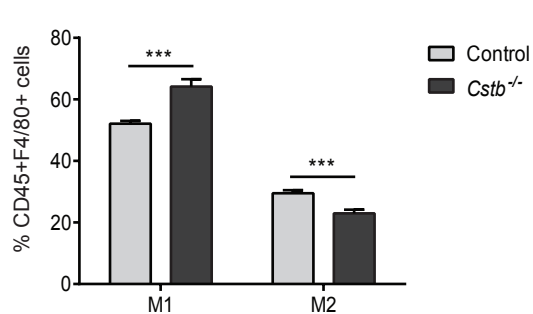**C****P14 brain**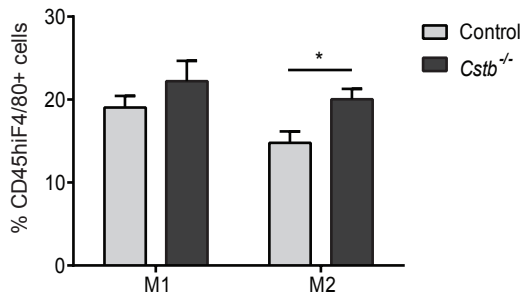**D****P30 brain**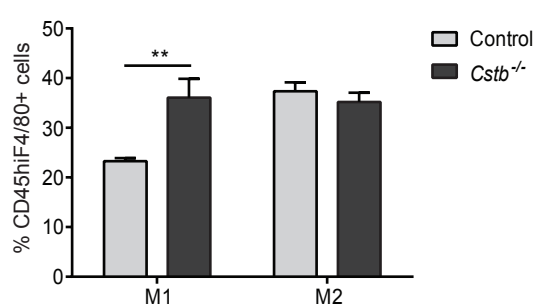

Supplement: Additional file 6: Figure S5. — Flow cytometric analysis of M1 and M2 macrophages in control and Cstb −/− mouse spleen and brain. Percentages of M1 and M2 macrophages in the total macrophage population in the (A and B) spleen and (C and D) brain of control and Cstb −/− mice at P14 and P30. (*p < 0.05, **p < 0.01, ***p < 0.001). (PDF 309 kb) [file 12974_2016_764_MOESM6_ESM.pdf]
